# Supplementary material for: Buddha image meditation is a potent predictor for mental health outcomes: A cross-sectional study among Thai high-school students
Source: PLoS One. 2025 Jun 23;20(6):e0326432. doi: 10.1371/journal.pone.0326432 (PMC12184913; doi:10.1371/journal.pone.0326432)
Supplement: Attachment — Submitted filename: Response to Reviewers1.pdf [file pone.0326432.s002.pdf]

Dear editor and reviewers,

We appreciate your valuable comments and suggestions. Please see below our point-by-point responses.

1. Please ensure that your manuscript meets PLOS ONE's style requirements, including those for file naming. The PLOS ONE style templates can be found at https://journals.plos.org/plosone/s/file?id=wjVg/PLOSOne_formatting_sample_main_body.pdf and https://journals.plos.org/plosone/s/file?id=ba62/PLOSOne_formatting_sample_title_authors_affiliations.pdf

Response. We have checked our manuscript accordingly.

2. Please amend your current ethics statement to address the following concerns:
a) Did participants provide their written or verbal informed consent to participate in this study?

Response: Participants and guardians provided written consent. This was amended in the Materials and methods section and reads, "Written informed consent was obtained from participants and most of the participant's guardians. The school administrators provided written consent for participants whose parents were unable to be reached."

3. In your Data Availability statement, you have not specified where the minimal data set underlying the results described in your manuscript can be found. PLOS defines a study's minimal data set as the underlying data used to reach the conclusions drawn in the manuscript and any additional data required to replicate the reported study findings in their entirety. All PLOS journals require that the minimal data set be made fully available. For more information about our data policy, please see http://journals.plos.org/plosone/s/data-availability.

Response. We have attached the data file to this submission.

4. Please amend either the title on the online submission form (via Edit Submission) or the title in the manuscript so that they are identical.

Response: the title in the manuscript has been updated in line with the online submission form. "Buddha image meditation is a potent predictor for mental health outcomes: a cross-sectional study among high-school students."

5. Please include your full ethics statement in the 'Methods' section of your manuscript file. In your statement, please include the full name of the IRB or ethics committee who approved or waived your study, as well as whether or not you obtained informed written or

verbal consent. If consent was waived for your study, please include this information in your statement as well.

Response: the full name of the ethics committee and that the participants provided written consent was included in the Materials and methods section and reads, "Written informed consent was obtained from participants and most of the participant's guardians… This study was approved by the Research Ethics Committee, Faculty of Medicine, Chiang Mai University, Thailand. The study code is 236/2021 and the date of approval was 10 June 2021."

Reviewers' comments:

Reviewer's Responses to Questions

Comments to the Author

Reviewer #1: 1. Experiments, statistics, and other analyses are performed to a high technical standard and are described in sufficient detail.
a. More details on recruitment and data collection are needed (e.g., when was data collected, paper or electronic surveys, completed in school or at home, etc.).

Response: Data collection is specified in Materials and methods section - between July and Aug 2021. Information was provided about survey type and location of completion in Methods and materials section, "Data collection was conducted with both electronic and paper surveys when the students were at their schools."

b. Under Materials and Methods (p. 5) – how was quality of meditation practice measured using the MEQ? Only questions regarding meditation styles and frequency are described.

Response: The remark about quality was deleted.

c. Under Materials and Methods (p. 5-6) – for consistency, please include Cronbach's alphas for all instruments used (it was reported for RI-9 and PSS-10 but not the other measures).

Response: Cronbach's alphas were included in Instruments section for RSES α=0.80, OI-anxiety subscale α=0.83, and OI-depression subscale α=0.76.

d. Please provide more information on how imputation was performed, i.e., what data were used to impute the missing values.

Response: Expectation Maximization (EM) imputation was used to fill in the randomly missing data.

e. There is no mention of ANOVA in the Statistical Analysis section (p. 7), but results from an ANOVA are later described under Results and Discussion (p. 10). Please include a description of the ANOVA performed in the Statistical Analysis section.

Response: the section Statistical Analysis has been edited to include mention of ANOVA. "The association between demographic data, style of meditation, and mental health outcomes was assessed using analysis of variance (ANOVA) and Pearson's and biserial correlations."

f. Under Results and Discussion (p. 7) it says "Ten outliers were excluded from the final data" – what criteria were used to determine outliers? This should be noted in the Statistical Analysis section.

Response: Ten outliers were identified in the regression analysis; the residual and distance values were calculated. The cases suggesting outliers were from the final data.

g. Why were regressions not estimated for the other meditation types, i.e., those not included in the paper? Why were some demographic variables not included in the regressions?

Response: The other meditation styles were included in the stepwise regression, yet they were found to be non-significant at every step in the process.

2. Conclusions are presented in an appropriate fashion and are supported by the data.
a. "A small correlation between kasina and practice frequency may explain the trend" (p. 12) – please explain this further. Has this been demonstrated in past research?

Response: This remark was expanded and a citation provided that reads, "A small, significant correlation between *kasina* and practice frequency (Table 2) may explain the trend toward significance as regular meditation practice was found to positively influence mental health in other research {Basso, 2019, Brief`, Daily Meditation Enhances Attention`, Memory`, Mood`, and Emotional Regulation in Non-Experienced Meditators}."

b. "Mindfulness meditation practice is common in Thailand, but it is not often practiced by adolescents in northern Thailand" (p. 13) – please explain this further. Has this been demonstrated in past research?

Response: This sentence was deleted.

c. "Among all styles of meditation, only Buddha image visualization, Manomayiddhi, and breathing meditation have proved to be predictors of mental health" (p. 13) – in the current sample yes, but the way it is worded makes it sound like you are referring to this generally, which is not accurate.

Response: This sentence was edited to be more clear, "Among seven types of meditation analyzed for, only Buddha image visualization, *Manomayiddhi,* and breathing meditation proved to be predictors of mental health."

d. Second paragraph on p. 13 – it would make more sense to discuss the results for each style in the order they are listed in that first sentence ("Among all styles of meditation, only Buddha image visualization, Manomayiddhi, and breathing meditation have proved to be predictors of mental health"). Consider reorganizing this section so that Buddha visualization is discussed first, then Manomayiddhi, and then breathing meditation.

Response: this recommendation is appreciated, and it has been implemented.

e. Last paragraph on p. 14 – please elaborate on why Buddha image visualization and Manomayiddhi are mostly practiced at Buddhist boarding schools. You make this statement, but the following sentences don't necessarily connect back to this point.

Response: Indeed, more information was provided regarding this important point, "In this sample, correlation results highlight that Buddha image and *manomayiddhi* are practiced more frequently at Buddhist boarding schools and not at secular schools, highlighting how school types differ in relation to meditation practice. School type was also found to be a significant influence in the resilience regression model. Buddhist boarding schools are often connected to a Buddhist temple that designates monks to instruct the students, a factor which can influence the quality of meditation instruction. Secular schools can lack temple affiliation and may not emphasize meditation as part of the curriculum. Additionally, regional differences in training styles can elevate the practice of certain meditation types over others."

f. "Data regarding negative experiences during meditation were not collected, a relevant consideration as perceived stress scores indicated infrequent or everyday meditation may be a stressor" (p. 16) – please elaborate on this. Has this been seen in previous research? Further discussion on these findings is warranted.

Response: this sentence was removed.

g. In the Conclusion section (p. 16), please provide additional context for these results (e.g.,

"This research demonstrates that many types of meditation yield varied results among adolescents in Thailand").

Response: more context was added about these results, "This research demonstrates that meditation type yields varied mental health results among Thai boarding school adolescents."

3. The article is presented in an intelligible fashion and is written in standard English.
a. In general, the writing can be difficult to follow and switches between past and present tense.

4. The research meets all applicable standards for the ethics of experimentation and research integrity.
a. Yes, the authors indicate the study received approval from an ethics committee and informed consent was obtained from participants.

5. The article adheres to appropriate reporting guidelines and community standards for data availability.
a. Under Data Availability, the authors state that some restrictions will apply but then below state that the data underlying the findings are fully available without restriction. Please clarify.

Response: the minimal data set has been made available.

Other notes:
6. Introduction
a. I think it would be beneficial to elaborate on mental health issues among adolescents in Asia during/after COVID-19 lockdowns, as well as any past research on meditation-based interventions in schools in Thailand.

Response: Information about adolescents in Asia has been clarified in the introduction, "In addition to increasing self-esteem, meditation has also been linked with increased resilience [8, 9], an important quality for coping with stress, anxiety, and depression, which are commonly reported mental health issues in adolescents during secondary education [10-12] and later adolescence [13]. The prevalence of these mental problems was exceptionally high amongst children and adolescents in Asia following the COVID-19 lockdown experiences [14, 15]."

b. Has past research examined the mental health benefits of Kasina, Buddha image visualization, and Manomayiddhi? If so, please include that in the introduction in t.

Response: Buddha Image and Manomayiddhi have not been previously researched, and mention of this has now been included in the Meditation Types section. Kasina has had previous mention. Please refer to the meditation style section for this information.

7. Overall

a. There seems to be a large overlap in the content of these different meditation styles, e.g. Buddha image is part of Manomayiddhi, so these results do not help us understand what components of Manomayiddhi may be helpful.

Response: This is an important point. More clarity was brought to how these styles are integrated in practice. “In the case of *manomayiddhi*, meditators will generally use Buddha image visualization, however; not all Buddha image meditators practice *Manomayiddhi* (Table 1 for practitioner totals). Buddha image visualization can be a stand-alone technique, but it is often practiced for focus prior to *manomayiddhi* and can be integrated within. This is similar to breathing meditation followed by vipassana and occasionally switching back to breathing meditation as necessary.”

b. There is no discussion of why some styles might not be associated with better mental health.

Response: consideration of why some styles might not be associated with better mental health has been included. “However, the integration of the aforementioned types of meditation into *manomayiddhi* raises questions about why recollections, vipassana, mindfulness, and *kasina* were not significant predictors in the regression models. Some explanation for these styles’ non-significant results may be explained by consideration of the quality of instruction. In this sample, correlation results highlight that Buddha image and *manomayiddhi* are practiced more frequently at Buddhist boarding schools and not at secular schools, highlighting how school types differ in relation to meditation practice. School type was also found to be a significant influence in the resilience regression model. Buddhist boarding schools are often connected to a Buddhist temple…”

Reviewer #2: Thank you for giving me the opportunity to read and comment on this paper. I find the topic very interesting. I thank the authors for their efforts at these times. Having said that, I suggested some comments which should be addressed.

1. The abstract section needs more changes, as the main results did not well articulate. Pay attention to reporting the main results of the study, as the results in the abstract do not convey the main findings of your study. The background paragraph in "Abstract" must be deleted and changed. The background section should be the shortest part of the abstract and should very briefly outline: What is already known about the subject, is related to the paper in question. What is not known about the subject and hence what the study intended to examine??

Response: An abstract which better reflects the research has been included,

“***Purpose:*** Meditation has been demonstrated to benefit adolescent mental health. However, less-known meditation styles have received sparse research attention. This research

examined various meditation styles in northern Thailand to determine which were associated with positive and negative mental health outcomes in adolescents.

Population and Methods: Boarding school students, 15-18 years, from secular and Buddhist schools in Northern Thailand provided information about meditation styles and frequency during the last month (i.e., breathing, *kasina (color)*, Buddha image visualization, *manomayiddhi*, mindfulness, recollections, and vipassana). The Rosenberg Self-Esteem Scale (RSES), Resilience Inventory (RI-9), Outcome Inventory-21 (OI-21), and Perceived Stress Scale (PSS-10) were completed. Stepwise regression was used to identify the effects of meditation styles on mental health outcomes.

Results: Among 443 participants, 390 were females (87.9%). The mean age was 16.35 (0.96) years. The three most common meditation styles practiced were breathing, Buddha image visualization, and mindfulness (46.5%, 26.2%, and 22.8%, respectively). Buddha image visualization was a significant ($p < .001$) predictor of RSES (β =.18), RI-9 (β =.22), OI-Anxiety (β = -.23), OI-Depression (β = -.26), and PSS-10 (β = -.20), whereas *manomayiddhi* was a predictor of RI-9 (β= .165, p =.001), OI-Anxiety (β = -.20, $p < .001$), OI-Depression (β = -.18, $p < .001$), and PSS-10 (β= -.154, p = .002). Breathing meditation predicted OI-Depression (β= -.129, p =.004). Daily meditation frequency was associated with the best mental health scores ($p <.001$).

Conclusion: Buddha image visualization, *manomayiddhi*, and breathing meditation were predictive of adolescents' mental health. A higher practice frequency is associated with positive mental health outcomes.

2. 3. The introduction (background) here is not adequate. The background of your study did not provide context to the information discussed throughout the research paper. The background should include both important and relevant studies on meditation and it's associated with general health and mental health outcomes. This is particularly important to support your paper.

Response: This was addressed with the addition in the second paragraph, "This study seeks to determine the relationship between meditation and mental health amongst adolescents in northern Thailand." and subsequent addition of more context leading into Meditation Types section "There are many meditation styles practiced in Thailand and abroad, such as mindfulness and vipassana. Buddha Image and *Manomayiddhi* are two styles not known by this study's authors to be practiced outside of Thailand. The remaining styles are more common and discussed in Buddhist texts {Shaw, 2006, Buddhist Meditation: An Anthology of Texts from the Pali Canon}{Buddhaghosa, 2010, The path of purification (Visuddhimagga)}. Some meditation styles are not difficult to learn or practice, whereas other techniques require guidance." Otherwise, information about the mental health benefits of these meditation types is available in paragraph 2 (Meditation and Mental Health) and on page 4 onward in the specific meditation sections.

3. Please report the full questionnaire used in this study.

Response: We have provided sufficient information regarding the questionnaiires used in the study. Please specify if more information is necessary.

4. What is the non-response rate of the study? Moving forward, all reviewers will likely ask for a response rate. The study should state…

a. clearly how participant recruitment was performed - e.g. did study personnel reach out to participants, or some other mechanism? e.g. were email lists used?

   Response: A research assistant contacted the schools by phone and email with information about the study.

b. how many participants received the survey?

   Response: From the two Buddhist schools, 179 and 57 students were recruited, and from the three secular schools, 145, 6, and 56 students were recruited.

c. of those who received the survey, how many participants responded- completely (plus how do you define complete) or partially?

   Response: Not all participants submitted complete data. Information about participant missing data has been added and reads, "Overall, 3% of the data was found to be missing, and 344 out of 453 participants submitted surveys with missing data.

d. for your analyses, did you include complete or partial responses?

   Response: Yes, Based on Ethics, the participants were allowed to skip any questionnaire she/he did not want to answer. Therefore, a 100% complete response is unlikely. The participant's data was used if they completed 80% or more of the survey.

e. Please add about how you try to maintain the correctness and accuracy of the data during data collection.

   Response: Data was pooled into one spreadsheet and reviewed by all of the study's authors to ensure accuracy.

6. Please add how sample size was calculated.

Response: The total sample was originally calculated to accommodate SEM with a final sample of 453 participants. For this secondary data analysis, sample size calculation for linear regression was determined by the medium effect size, significance level (alpha) of 0.05, power (beta) of 0.8. This yielded a number of at least 114 required for analysis, with a final sample of 443 participants. The total sample was included for analysis.

7. Please report results and discussion in separate sections. The discussion was not adequate and need to mention related studies to provide readers with appropriate interpretations. Pay attention to mentioning more related studies with respect to your study. The discussion section is not backed up by a sufficient literature review. Recent references can be added to strengthen the argument of the impact of image meditation on mental health outcomes.

Response: The discussion section was revised, and more information was provided to contextualize the findings.

Hopefully, we have addressed all the reviewers' and the editor's concerns. We are looking forward to your reply.

Best regards,

Tinakon Wongpakaran
