# Supplementary material for: Buddha image meditation is a potent predictor for mental health outcomes: A cross-sectional study among Thai high-school students
Source: PLoS One. 2025 Jun 23;20(6):e0326432. doi: 10.1371/journal.pone.0326432 (PMC12184913; doi:10.1371/journal.pone.0326432)
Supplement: Attachment — Submitted filename: Response to Reviewers 3May2024.pdf [file pone.0326432.s006.pdf]

Dear Editor, Sally Mohammed Farghaly.

We appreciate the reviewers and the editor for providing us with an opportunity to improve our manuscript. Please see below our point-by-point response to the comments.

Reviewer #5: The authors of the paper, "Buddha image meditation is a potent predictor for mental health outcomes: a cross-sectional study among high-school students", structured the paper nicely covering both statistical theories on a multiple linear regression model analysis and its application to examine meditation styles and mental health outcomes. I found the paper contributes little to either statistical modeling or meditation styles and mental health outcome literature. I found some issues discussed below:

Response. Thank you very much for the feedback. We hope to have addressed all of the reviewer's points. For the reviewer's information, the authors have revised one remark and included additional mention about this paper's purpose at the end of the intro and discussion to better highlight how this paper contributes to research on meditation styles. The sentence in the intro was edited and now reads, "This study seeks to determine the relationship between different meditation styles and mental health outcomes of adolescents in northern Thailand." Additionally, a sentence was added to the end of the discussion that reads, "Moreover, this survey of different meditation styles practiced across northern Thailand indicates some meditation types are more impactful on mental health than others. Further study on less researched meditation types, particularly death recollections, Buddha image, and *manomayiddhi*, are warranted."

1. In the methods:
- You simply stated as you have used stepwise multivariable linear regression analysis models. You have not written the model (equation) in the method part even if the model name you wrote is not correct.
Response: We have added the model equation in the method part, and the incorrect titling of the method of analysis has been corrected throughout the paper (ie. abstract, methods, results).

How many models did you use for your data analysis?
Response. The number of models used was added to each of the results sections. For positive variables, "Both the self-esteem and resilience models used four iterations to arrive at the final model." For the negative variables, "Regarding the stepwise method used, four iterations were used in the anxiety model, with three iterations used in the depression and perceived stress models."

In Statistics, there is no stepwise multivariable linear regression analysis model. Please make correction on the model name. Say Multiple linear regression model analysis. I understand that for the selection of important variables/factors, you used stepwise variable selection method. Please refer materials on the model you used to assure whether you are right or not.
Response. Thank you, this has been corrected.

- How did you select two schools deliberately? No other schools?. (non probability convenient sampling because of covid for secular schools) Non-probability sampling

techniques such as purposive sampling, quota sampling, ...) are bias based sampling methods. It's not recommended to use such sampling methods unless a compelling situation arises

Response. The two Buddhist boarding schools were the only two Buddhist boarding schools in northern Thailand and both accepted. Three other secular schools also accepted. This purposive sampling method was employed in order to ensure that this study had sufficient meditators. Additionally, this study was conducted just as the Covid-19 pandemic lockdowns were going into effect. Many schools did not accept the invitation due to the pandemic, and this study accepted interested schools on a first-come first-serve basis.

2. In the results:
- Incorporate statistical findings, such as charts and graphs, into your content.

Response. Two images were included (Figure 1 and Figure 2) that depict meditation styles and meditation practice frequencies of this population.

We hope that our responses address the reviewers' concerns. We are looking forward to receiving your feedback.

Best regards,

TW
