# Supplementary material for: Buddha image meditation is a potent predictor for mental health outcomes: A cross-sectional study among Thai high-school students
Source: PLoS One. 2025 Jun 23;20(6):e0326432. doi: 10.1371/journal.pone.0326432 (PMC12184913; doi:10.1371/journal.pone.0326432)
Supplement: Attachment — Submitted filename: BuddhaImage_Reviewer Replies.pdf [file pone.0326432.s011.pdf]

Dear Editor,

Based on the editor's comments, *"This was my earlier comment to you but there has not been a revision or rebuttal to the comment (actually from reviewer 8). I realised that you did not respond to the comment of Reviewer 8. Therefore, I will entreat you to respond to it.*

I do not know whether you may still see it therefore, I am stating it here: "The paper seems to be modified based the reviewers' comments.
I just have a one comments about mother's skills and their health literacy that can have a mediated role in promoting the mental health of the school child people. So I suggest to check the following paper for more conceptualization of the mentioned issue:

Enabling mothers through improving mental health literacy and parenting skills
Biosocial Health Journal. 2024;1(1): 26-32. doi: 10.34172/bshj.4"

Please ignore the comment of Reviewer 9 this time or any other comments from a reviewer. Only address the one above (repost from Reviewer 8)."

The editor's remarks about disregarding the reviewer's comments and considering the use of an unrelated manuscript on mental health literacy and parenting skills were already addressed **in the last round of reviews.** It is the second time the editor has sent that reply. Please consider that matter sufficiently addressed.

Comments to the Author

Reviewer #7: Review comments
General comments: I commend the Authors for their good understanding all through the period of the review of this manuscript. I recommend that the manuscript should be accepted for publication.
It is important that the use of square brackets for reference numbers is corrected before publication.

Thank you for your commendation and for recommending the acceptance of the manuscript. The square brackets have been added.

We believe we have addressed all comments. If there are any additional points for consideration, please let us know."

Best regards**,**

TW
