# Supplementary material for: Buddha image meditation is a potent predictor for mental health outcomes: A cross-sectional study among Thai high-school students
Source: PLoS One. 2025 Jun 23;20(6):e0326432. doi: 10.1371/journal.pone.0326432 (PMC12184913; doi:10.1371/journal.pone.0326432)
Supplement: Attachment — Submitted filename: Response to Reviewers_2.docx [file pone.0326432.s003.docx]

Dear Editor,

Thank you for providing us with an opportunity to improve our manuscript. Please see below our response to the reviewer

**Review Comments to the Author**

Reviewer #3 comment: The sample size calculation seems to have deviation with the recruited subjects into the study. Please clarify.

**Author’s response:**  In fact, no deviation occurred. However, the author’s previous response about sample information did omit important information relevant to your follow-up.

In the author’s previous response, we wrote, “The total sample was originally calculated to accommodate SEM with a final sample of 453 participants. For this secondary data analysis, sample size calculation for linear regression was determined by the medium effect size, significance level (alpha) of 0.05, power (beta) of 0.8. This yielded a number of at least 114 required for analysis, with a final sample of 443 participants. The total sample was included for analysis.”

The total sample of 453 participants had ten outliers removed for analysis with a final total of 443 participants. This information is found in the Results section and reads, “In total, 443 responses were included in the analysis out of 453. Ten outliers were identified in the regression analysis and excluded from the final data.”

We hope our clarification would satisfy the reviewer’s question. Should you have any further questions, please let us know. Thank you so much.

Best regards,

TW, JD, and all
