# Supplementary material for: Buddha image meditation is a potent predictor for mental health outcomes: A cross-sectional study among Thai high-school students
Source: PLoS One. 2025 Jun 23;20(6):e0326432. doi: 10.1371/journal.pone.0326432 (PMC12184913; doi:10.1371/journal.pone.0326432)
Supplement: Attachment — Submitted filename: Response to Reviewers 7Nov23.docx [file pone.0326432.s004.docx]

Dear Editor

We greatly appreciate the reviewer's keen and insightful comments, which provide us with an opportunity to refine our manuscript. Below, you will find our detailed responses to each comment.

The study reported effects of different meditation style on mental health of high school students in Thailand. After reviewing, I have some comments as follows.

Abstract
REVIEWER: Please add the inclusion criteria for enrolling students into the study.

**AUTHOR:** Additional information about purposive selection was added to the abstract as follows.

Participants in this study encompassed high school students who were 15-18 years old, irrespective of their gender and religious background, and were enrolled in grades 10-12 in either secular or Buddhist Thai boarding schools.

Introduction
REVIEWER:-Could the authors provide more background information about the study setting. How many high school students (or general population) in Thailand practice meditation in daily life?

**AUTHOR:** Yes, that information will help with contextualization. Thank you. This sentence was added on page three in the first line of the Introduction/Meditation and Mental Health section. We have added the information as follows.

Meditation is a widespread practice in Thailand that spans across all age groups. However, there is a need for more comprehensive studies to accurately gauge the prevalence of meditation among high school students and the general population in the country. Nonetheless, it is a common ritual in schools to engage in meditation and chanting before class, and it's reasonable to expect that meditation practices are more prevalent and more structured in Buddhist schools compared to secular institutions. Additional studies have indicated that approximately 17% of the general Thai population, and as much as 58% of older residents, actively participate in meditation, highlighting a significant number of practitioners with a variety of meditation styles (1, 2).

REVIEWER: What are the prevalence of mental health among students in boarding schools?

Are they different from students who reside with biological family? Did the prevalence of mental health problems in students change during Covid-19 period?

**AUTHOR:** Answering the above three questions has helped to contextualize the research more. Thank you. This text was added to page 3 in the middle of the “Meditation and mental health section,”

“Previous pre-pandemic research revealed parity in the mental health of students from various schools; however, loneliness issues have been reported among boarders (15, 22). Studies conducted during the Covid-19 pandemic in Southeast Asian countries, including Thailand, have indicated that Thai adolescents reported significantly higher levels of stress, anxiety, and depression symptoms compared to their counterparts in other countries (17-20). Global estimates suggest that the prevalence of depression and anxiety doubled in response to the pandemic (21).

Methods
REVIEWER: -Could the authors describe more about inclusion criteria and recruitment process? How did those students come to join the study, if the authors did not include everyone in the school?

**AUTHOR:** Students were invited to join via an information session. The participating schools announced the research opportunity and interested students were invited to the information session. During this session the informed consent was read, and any questions were answered. Interested students were given approximately one week to speak with their families to ask for permission to participate. Information from this explanation was added to the “Materials and Methods” section on page 6 and reads,

“This research was an observation study. The study recruited 453 participants. The Inclusion criteria for the study were:

- Students who were 15 years old and older.
- Studying in grades 10-12.
- Studying in a Thai boarding school in Northern Thailand.
- Having access to a computer with internet.
- All religious groups.

The Exclusion criteria were students with special needs and students who were blind or deaf.

In the recruitment process, students were invited to join via an information session. The participating schools announced the research opportunity and interested students were invited to the information session. During this session the informed consent was read, and any questions were answered. Interested students were given approximately one week to speak with their families to ask for permission to participate. Since participation in this study was voluntary, not every student showed interest and chose to take part. The data collection period was between July and August 2021. The schools were purposively selected to reflect similar socioeconomic status, the number of students, and the female-to-male ratio. A research assistant contacted the schools by phone and email with information about the study. Two Buddhist schools in urban areas and three secular schools in urban and suburban areas from three provinces participated. An information session was provided to interested students to learn about the research, with data collection occurring the following week for those students willing to participate. From the two Buddhist schools, 179 and 57 students were recruited, and from the three secular schools, 145, 6, and 56 students were recruited. The variation in students willing to participate was likely due to data collection occurring amidst a lockdown announcement, with many students opting to return home opposed to sheltering at their boarding school. Students with special needs and students who were blind or deaf were excluded. Written informed consent was obtained from participants and most of the participants’ guardians. This study was approved by the Research Ethics Committee, Faculty of Medicine, Chiang Mai University, Thailand. Study (code 236/2021) and approved on 10 June 2021. A related study was published elsewhere (43).

REVIEWER: The number of students varied from one school to another, how they were selected?

**AUTHOR:** Yes, the number of students willing and able to participate varied due to a covid-lockdown announcement that coincided with the data collection. Many students were returning home, though some remained in the schools. The selection process for students was voluntary. Additional information about this was added to the “Materials and Methods” section on page 6 and reads, “The schools were purposively selected to reflect similar socioeconomic status, the number of students, and the female-to-male ratio. A research assistant contacted the schools by phone and email with information about the study. Two Buddhist schools in urban areas and three secular schools in urban and suburban areas from three provinces participated. An information session was provided to interested students to learn about the research, with data collection occurring the following week for those students willing to participate. From the two Buddhist schools, 179 and 57 students were recruited, and from the three secular schools, 145, 6, and 56 students were recruited. The variation in students willing to participate was likely due to data collection occurring amidst a lockdown announcement, with many students opting to return home opposed to sheltering at their boarding school. Students with special needs and students who were blind or deaf were excluded.

REVIEWER-There were six students from a secular school, would they be a good representative of students from their school?

**AUTHOR:** The six students were willing to participate in those that remained at the school just prior to the Covid-19 lockdown. The six-student total is not intended to be representative of the school, and the authors could not justify excluding the participants due to the low number.

REVIEWER-Did the authors explain about different types of meditation to potential study participants?

**AUTHOR:** No explanation about meditation types was discussed. Participants were able to mark on the meditation questionnaire if they did not meditate. Names of meditation types known to be practiced were listed on the questionnaire to be marked if practiced. A space was provided to write in additional types if they were practiced.

REVIEWER: Did they have a chance to learn how each type was?

**AUTHOR:** No education about the different types was provided. Meditation is freely available to be practiced at temples all over Thailand and through many schools. The style of meditation listed is standard for general Thai people. This research sought to learn what meditation styles the students had practiced, and 88 % practiced at least one of those styles.

REVIEWER: Were there any interventions offered before, during, or after the study period?

**AUTHOR:** No interventions were provided. This research was an observation study and sought to understand which meditation styles were practiced amongst adolescents and how often during the last month.

REVIEWER:-How was the data collected? (by paper-based questionnaires, online, face-to-face interview, or other methods)

**AUTHOR:** Data was collected both online and with paper-based forms, depending on participants' preferences and the availability of time and equipment. Information about this is available in the Materials and Methods section and reads, “Data were collected using both electronic and paper surveys when the students were at their schools.”

Results
REVIEWER-There were 10.6% of students who were not Buddhist, what were their religions and why they were included?

**AUTHOR**-The 10.6% comprised minority religious groups and non-religious participants. These participants were categorized as non-Buddhist together. This population was included as these participants may still choose to meditate in their schools or at temples. A minority religious status or non-religion holder does not mean they are excluded from these practices in Thailand. Additionally, the participants were able to mark if they did not practice meditation.

REVIEWER: If they were Christian or Muslim, they might not be comfortable looking at the Buddha mage or understand those meditation style names. How were the questions asked?

**AUTHOR:** The form stated that this questionnaire was to be about meditation, and that participants can fill it out whether they have or have not meditated in the last month. If they had meditated, mark how frequently meditation occurred. Then the styles practiced in the last month were listed and the participants were instructed to mark any of them or to fill in additional types if relevant. Given that contemplating on a Buddha image is a common practice in Thailand, we have identified it as a separate variable. This allows individuals who meditate on images other than that of Buddha, such as non-Buddhists, to choose "other options" and provide additional details.

We have added this part in **Meditation Evaluation Questionnaire (MEQ).**

REVIEWER:-Did the authors collect data on their lifestyle, social behaviors, or baseline mental health before meditation practice (by self-report, teacher report, or school record etc.)

**AUTHOR:** Data was collected on mental health via self-report during that day. Additional questionnaires were provided regarding lifestyle and social behaviors, though that information was not included in this secondary data analysis.

No, we did not gather the data you mentioned "prior to" the participants' meditation sessions as this was not an intervention study. However, the questions related to lifestyle and social behaviors were tailored to the respective questionnaires. For instance, questions about meditation and precepts focused on the past month, while inquiries about other mental health issues pertained to the past two weeks. Additionally, their baseline mental health information was collected in the form of their past history concerning physical and mental health.

We hope our response will sufficiently address the reviewers’ concerns. We are looking forward to hearing from you soon.

Best regards,

JD, TW, NW and DW.
