# Supplementary material for: Buddha image meditation is a potent predictor for mental health outcomes: A cross-sectional study among Thai high-school students
Source: PLoS One. 2025 Jun 23;20(6):e0326432. doi: 10.1371/journal.pone.0326432 (PMC12184913; doi:10.1371/journal.pone.0326432)
Supplement: Attachment — Submitted filename: Review comments.docx [file pone.0326432.s005.docx]

**Review comments**

**General comments**

I thank the Editor for the opportunity to review this manuscript which made an interesting read. Also, the findings from the study are of relevance. In reading the manuscript I made the following observations. Responding to these comments may ensure that the quality of the manuscript is improved.

1. The selection of the school was based on purposive sampling and there was no probability sampling technique in selecting the respondents. The Buddhists constituted 89.4% of the respondents. Does it mean that 10.6% of the respondents did not apply any meditation style and if so, were they included in the analysis of the data? Moreover, the Authors indicated that some respondents selected more than one meditation style. How was this taken into account in analyzing and interpreting the findings of the study.
2. The title of the study connotes a positive alignment towards ‘**Buddha image meditation.**’ A more expressive title should have been; Predictors of good or positive mental health outcomes among high school students in Thailand: a cross-sectional study. In any case it will be good to find out how the Authors adopted the title as presented in the manuscript.
3. Validated tools. It will be good if the Authors provide a link to each of the validated tools used in the study. The Authors reported that the Rosenberg self-esteem scale has a total score of 40. The score of the Rosenberg self-esteem scale ranges from 0-30. Authors should cross check the tool again for details.
4. An insight into the iterations as applied in the study should be provided to guide the readers on how the final regression model was obtained. Limiting the variables in the biserial correlation table may be necessary to enhance understanding of Readers.
5. There was no indication on how the sample size for the study was obtained. Authors indicated that the **schools were purposively selected to reflect similar socio-economic status, the number of students and the female to male ratio.** This was different from the response provided in the answer to a comment from a Reviewer. It will be important to know whether the purposive selection achieved its purpose.
6. The linear regression result in the abstract should include 95% confidence interval and p value deleted. The conclusion in the abstract section is simply a repetition of study findings and should be reviewed.
7. The sub-titles as applied in the Introduction section should be deleted. The section should be organized into distinct paragraphs. The objectives of the study should come at the end of the Introduction section of the manuscript.
8. There was no description of the study area. There was no indication on how the questionnaire was administered to the respondents especially since the Authors indicated that there were missing variables in the limitation of the study.
9. There is no need to include p values and table numbers in the discussion section of the manuscript. The subtitle named ‘limitation and future directions’ should be deleted.
10. Authors should ensure that use of references and referencing style as used in the manuscript conform to the requirements of the Journal.
