# Supplementary material for: Buddha image meditation is a potent predictor for mental health outcomes: A cross-sectional study among Thai high-school students
Source: PLoS One. 2025 Jun 23;20(6):e0326432. doi: 10.1371/journal.pone.0326432 (PMC12184913; doi:10.1371/journal.pone.0326432)
Supplement: Attachment — Submitted filename: Response to Reviewers 4Oct.docx [file pone.0326432.s008.docx]

**Dear Editor,**

We have reviewed the feedback and addressed all the comments. Additionally, we have checked the grammar and made edits for better clarity and understanding. Please see our point-by-point response to the comments below.

1. The selection of the school was based on purposive sampling and there was no probability sampling technique in selecting the respondents. The Buddhists constituted 89.4% of the respondents. Does it mean that 10.6% of the respondents did not apply any meditation style and if so, were they included in the analysis of the data? Moreover, the Authors indicated that some respondents selected more than one meditation style. How was this taken into account in analyzing and interpreting the findings of the study.

Thank you for your questions. You are correct that by purposively selection this sample was not gathered with a probability sampling technique. Information about this was added to the Limitations section and now reads, “School participation in this study lacked randomization due to purposive selection of two Buddhist boarding schools. Participants were also not randomly selected, limiting how these results can be generalized.”

Of the respondents, 10.6% marked that they did not meditate and these participants were included in the analysis. Of this sub-population of non-meditators (50 total), 38 were Buddhist religion and 12 were not Buddhist. Within the total sample (443), there were 47 participants who were non-Buddhist. It is the case that some non-Buddhist participants did meditate and some Buddhists did not meditate. All religions were included in this study. The questionnaires ask about the participant’s meditation practice in the last month. Information about the number of meditators that practice more than one style was added to the Figure 1 information about meditative styles practiced and reads as, “It was found that 132 participants practiced more than one meditation style in the last month.”

Regarding accounting for the influence of multiple meditation styles within the analysis, all meditation styles were included in the analysis. The regression results identified the strongest associations iteratively (stepwise regression) between meditation type and mental health outcomes. What may not be accounted for in the analysis is how meditation types have influenced one another (if at all). Information about this is included in the discussion section about meditation types and reads as, “Buddha image visualization can be a stand-alone technique, but it is often practiced for focus prior to manomayiddhi. This is similar to meditators who begin with breathing meditation before they practice vipassana. This interaction of meditation types suggests one type may influence another, and this is important to account for in controlled studies. It can be the case within religious schools in Thailand that Manomayiddhi is taught with a guide for novice meditators, which can elevate the quality of the practice beyond unguided meditation. It also can mean that Buddha image meditation may be guided if used prior to guided manomayiddhi. It may be that the instruction benefits Buddha image practice, and differences in outcome might be found between guided and non-guided Buddha image and manomayiddhi meditators.” Information about the influence of meditation types on one another was also added to the limitations section and reads as, “Regarding meditation practice, the participants were able to select more than one meditation practice without indicating why a given meditation was preferred over others, how frequently each meditation was practiced, and if meditation types were combined. For example, breathing meditation may be practiced for five minutes before vipassana meditation begins. That information, along with other qualitative information, would help in evaluating the accessibility, difficulty, and benefits of these meditation types.” In addition, information was added to the conclusion about mixing of meditation types in order to encourage detailed accounts of the mixing of types and influences they may have on one another. This addition reads as, “This research also highlights the complexity of observational studies about meditation particular to the use of mixing meditation techniques. Future research on meditation types should include practice frequency, detailed documentation of meditator experiences, as well as the factors associated with meditation’s effects on mental health issues.”

2. The title of the study connotes a positive alignment towards ‘**Buddha image meditation.**’ A more expressive title should have been; Predictors of good or positive mental health outcomes among high school students in Thailand: a cross-sectional study. In any case it will be good to find out how the Authors adopted the title as presented in the manuscript.

Thank you for questioning the use of this particular title. The title was formed in response to the study's results, which indicated that Buddha Image meditation was found to be the most influential of the meditation styles included in the model for this population. The use of ‘potent’ might be perceived as a positive alignment. However, it is more specifically based upon the observation that the Buddha Image is the most efficacious of the meditation types analyzed on mental health.

3. Validated tools. It will be good if the Authors provide a link to each of the validated tools used in the study. The Authors reported that the Rosenberg self-esteem scale has a total score of 40. The score of the Rosenberg self-esteem scale ranges from 0-30. Authors should cross check the tool again for details.

Thank you. For the RSES scoring, the original RSES did use score ranges from 0-30. Other scoring options can be used, such as 10-40. The authors have revised the text in the RSES instrument section to clarify the point that the authors of the Thai version used the 10-40 scoring option. It now reads, “The Rosenberg Self-Esteem Scale (39) is used internationally to measure self-esteem. The reliability and validity of the tool has had extensive testing in various languages and was found to be effective. A revised version of the tool was tested with Thai adolescents and found to be equally reliable but with better construct validity (40). The RSES is brief and easily administered, utilizing a Likert-scale with ten items answered on a four-point scale that range from strongly disagree (1) to strongly agree (4), with half of the items having positive wording and half having negative wording. The total scores for the Thai version range from 10 to 40 with higher scores indicating higher self-esteem. The scale was tested with this study’s participants and resulted in a Cronbach’s α of 0.80.”

4. An insight into the iterations as applied in the study should be provided to guide the readers on how the final regression model was obtained. Limiting the variables in the biserial correlation table may be necessary to enhance understanding of Readers.

Thankyou, more information was added explaining the iterative process and how many iterations the different models used. It reads as, “. Multiple linear regression model analysis using the stepwise method for selective variables was used to determine the association along with other covariates including age, sex, and school type, as they were potential confounders. Stepwise regression is an iterative process of adding and removing variables based on their statistical significance and contribution to the model.” Within the results sections, more information about how many iterations occurred was included and reads as, “Table 4 is a multiple linear regression analysis of positive mental health outcomes, having used the stepwise method for selecting variables. Both the self-esteem and resilience models used four iterations to arrive at the final model.” And “Table 5 accounts for the influence of meditative styles on negative mental health outcomes. Regarding the stepwise method used, four iterations were used in the anxiety model, with three iterations used in the depression and perceived stress models.”

Regarding the bivariate correlation chart, the authors see the wisdom of reducing the number of variables in order to help the readers understand the most relevant variables more. Within the bivariate chart, Vipassana meditation and Mindfulness meditation were removed due to limited significant associations. The Table 2 text now includes the significant associations those two meditation styles did produce and reads as, “Vipassana meditation was significantly associated with kasina meditation .199, p<.01. Mindfulness meditation was significantly associated with kasina meditation .204, p<.01 and recollection of death meditation .192, p<.01. Both Vipassana and Mindfulness meditation were not included in the correlation chart (Table 2) due to the limited number of significant results.”

5. There was no indication on how the sample size for the study was obtained. Authors indicated that the **schools were purposively selected to reflect similar socio-economic status, the number of students and the female to male ratio.** This was different from the response provided in the answer to a comment from a Reviewer. It will be important to know whether the purposive selection achieved its purpose.

Thank you. Information about the sample size was included and reads as, “The sample estimation was originally calculated for structural equation modeling (SEM) with a final sample of 453 participants. For this secondary data analysis, a sample size calculation for linear regression was determined by the medium effect size, significance level (alpha) of 0.05, power (beta) of 0.8. This yielded a number of at least 114 required for the analysis. The total sample of 453 was included.”

Thank you for identifying this discrepancy between responses to reviewers. The information to both reviewers was correct, but not all of it was accounted for in the manuscript. This information has now been included and reads as, “The schools were purposively selected to reflect similar socioeconomic status, the number of students, and the female-to-male ratio. Buddhist boarding schools were prioritized when contacting schools to ensure a sufficient number of meditators participated.”

6. The linear regression result in the abstract should include 95% confidence interval and p value deleted. The conclusion in the abstract section is simply a repetition of study findings and should be reviewed.

Thank you for identifying this. The authors have deleted the p-values and stated that the regression results shown were significant.

Regarding the conclusion, the authors have included more information the reader may benefit from about this study. The conclusion now reads as, “This research demonstrates that meditation type yields varied mental health results among Thai boarding school adolescents. While mindfulness, kasina, vipassanā, and recollections meditations were not significant, Buddha image visualization, manomayiddhi, and breathing meditations were found to be effective and significant predictors using both positive and negative indices of mental health. As anticipated, frequent meditation practice predicted better mental health outcomes. This research may be the first to have assessed how Buddha image and manomayiddhi influence mental health, demonstrating gaps about meditation techniques that warrant exploration in future studies. This research also highlights the complexity of observational studies about meditation particular to the use of mixing meditation techniques. Future research on meditation types should include practice frequency, more detailed documentation of meditator experiences, as well as the factors associated with meditation’s effects on mental health issues.”

7. The sub-titles as applied in the Introduction section should be deleted. The section should be organized into distinct paragraphs. The objectives of the study should come at the end of the Introduction section of the manuscript.

Thank you. Sub-titles were deleted and distinct paragraphs were formed.

8. There was no description of the study area. There was no indication on how the questionnaire was administered to the respondents especially since the Authors indicated that there were missing variables in the limitation of the study.

Thank you. Additional information about the study area and the method for administering the questionnaire was added to the methods section and now reads as, “Boarding schools in northern Thailand were purposively selected to reflect similar socioeconomic status, the number of students, and the female-to-male ratio. Buddhist boarding schools were prioritized when contacting schools to ensure a sufficient number of meditators participated. A research assistant contacted the schools by phone and email with information about the study. Two Buddhist schools in urban areas and three secular schools in urban and suburban areas from three provinces participated. In the recruitment process, participating schools announced an information session that interested students could attend to learn about the research, with data collection occurring the following week. During this session, the informed consent was read and any questions were answered. Interested students were given approximately one week to speak with their families to ask for permission to participate. Since participation in this study was voluntary, not every student showed interest and chose to take part. The data collection period was between July and August 2021. From the two Buddhist schools, 179 and 57 students were recruited, and from the three secular schools, 145, 6, and 56 students were recruited. The questionnaires were administered both online and with paper depending upon the needs of the participating schools. The variation in students willing to participate was likely due to data collection occurring amidst a lockdown announcement, with many students opting to return home as opposed to sheltering at their boarding school.”

9. There is no need to include p values and table numbers in the discussion section of the manuscript. The subtitle named ‘limitation and future directions’ should be deleted.

Thank you, the p-values have been removed. The subtitle was deleted.

10. Authors should ensure that use of references and referencing style as used in the manuscript conform to the requirements of the Journal.

Thank you. The referencing style corresponds with the journals preferred citation style Vancouver.

We hope these revisions meet the expectations of the editor and reviewers. We look forward to hearing from you soon.

Best regards,

TW and colleagues
