# Supplementary material for: Buddha image meditation is a potent predictor for mental health outcomes: A cross-sectional study among Thai high-school students
Source: PLoS One. 2025 Jun 23;20(6):e0326432. doi: 10.1371/journal.pone.0326432 (PMC12184913; doi:10.1371/journal.pone.0326432)
Supplement: Attachment — Submitted filename: Review comments_07Feb (1).docx [file pone.0326432.s009.docx]

**Review comments**

**General comments**

I commend the Authors for the responses provided to the review comments as submitted during the review of the manuscript. I am of the opinion that the manuscript has some merits. I will endeavor to point out some comments that were not taken care of from the previous review comments.

Thank you for your helpful feedback. It was warmly received and appreciated.

Authors should take note of the following observations;

1. The title of the study connotes a positive alignment towards ‘**Buddha image meditation.**’ My observation is that the findings related to Buddha image meditation remarkably influenced the writing style of the Authors and re-phrasing the title may entail almost a re-writing of the manuscript. Based on this comment, I will allow the title to be except if the Editor thinks otherwise.

Thank you for sharing your perception of how the title can be interpreted. The authors’ suspect it was the use of ‘potent’ in the title that may have influenced the reviewer to view the title as positively aligned. It is not the case that ‘potent’ conveys positive alignment, only that the object (Buddha image meditation) is the strongest or most effective as an influence for the outcomes. If any other meditation type had been found to be the most effective, potent could still have been selected to convey the largest influence. The manuscript contents convey both that Buddha image meditation was the most effective predictor as well as that the meditation practices are embedded with religious influence. It is the author’s opinion that the content remains scientifically sound and educational about the cultural and religious understanding embedded within these practices.

2. There was no description of the study area. There was no indication of how the students were recruited into the study. (This should also be indicated in the abstract). There was no indication on how the questionnaire was administered to the respondents especially since the Authors indicated that there were missing variables in the limitation of the study.

Thank you for drawing the author’s attention to these points. Regarding study area, ‘northern Thailand’ is highlighted as the area of the study. However, the author’s have added the names of the provinces which the participating boarding schools were located in to provide more information (Chiang Mai, Lamphun, Uthai Thani, Phitsanulok). Regarding recruitment information and administration of the questionnaires for participants in the study, please find this in the methods section that reads, “A research assistant contacted the schools by phone and email with information about the study…. In the recruitment process, participating schools announced an information session that interested students could attend to learn about the research, with data collection occurring the following week. During this session, the informed consent was read and any questions were answered. Interested students were given approximately one week to speak with their families to ask for permission to participate. Since participation in this study was voluntary, not every student showed interest and chose to take part. The data collection period was between July and August 2021….The questionnaires were administered both online and with paper depending upon the needs of the participating schools. The variation in students willing to participate was likely due to data collection occurring amidst a lockdown announcement, with many students opting to return home as opposed to sheltering at their boarding school. Students with special needs and students who were blind or deaf were excluded. Written informed consent was obtained from participants and most of the participants’ guardians. The ethics committee approved the schools to provide permission for students whose parents were unable to be reached.” During the process of collecting data, it was found that 3% of the data was missing. This can be due to reasons such as accidentally or intentionally skipping a question. Participants were not required to complete all questions to avoid coercion for items that might make them uncomfortable. Both paper and pencil as well as online forms were used.

3. There is no need to include p values and table numbers in the discussion section of the manuscript.

Thankyou, the authors agree with this and have removed those details from the discussion.

4. Authors should ensure that use of references and referencing style as used in the manuscript conform to the requirements of the Journal. I encourage the Authors to visit the Journal website for guidance. For example, PLOS ONE makes use of block paragraphing and also prefer **square brackets for reference numbers.**

Thank you, the authors agree and have implemented the paragraph blocking. The in-text citation brackets are proving difficult at the moment. The authors will prepare for this change.

5. Ethical approval. The mean age of the students was recorded as 16.35±0.96 years. However, there was no indication that ‘assent’ was obtained from the respondents before including them in the study.

Information on informed consent can be found in the methods section and reads as, “…During this session, the informed consent was read and any questions were answered. Interested students were given approximately one week to speak with their families to ask for permission to participate…. Written informed consent was obtained from participants and most of the participants’ guardians. The ethics committee approved the schools to provide permission for students whose parents were unable to be reached.”

6. In the comments related to study limitation, ‘randomization and randomly selected’ may not be appropriate. The point to note is that probability sampling techniques were not used in the selection of the schools and in recruiting the students into the study.

Thank you. The author’s agree that your suggested wording has merit and have adapted the limitations section to read as, “Schools and students were recruited for the study without the use of probability sampling techniques, such as with the purposive selection of two Buddhist boarding schools. Participants also joined following announcements at participating schools, rather than by randomly selecting participants from those schools.”

7. P values should be written as p<0.001 instead of p<.001 to avoid confusion. The table on gender should be complete bearing in mind the sample size indicated at the beginning of the table.

Thank you. The authors have implemented these changes.

**Reviewer #9**

The formulation of the research objectives is also a point that deserves attention. The expression “This study seeks to determine the relationship between different meditation styles and mental health outcomes of adolescents in northern Thailand” could be revised to more accurately reflect what the study aims to achieve. The verb "determine" suggests a more rigid and definitive approach, while a verb such as "evaluate" or "understand" might be more appropriate

Thank you for your review of the manuscript and commentary. The authors have endeavored to improve the quality of the work along the lines of the reviewers remarks. Specifically, related to the specific wording used in the ‘objectives,’ the authors have edited the statement according to the reviewer’s remarks. The new objective reads as, “This study seeks to *understand* the relationship between different meditation styles and mental health outcomes of adolescents in northern Thailand.”
