# Supplementary material for: Buddha image meditation is a potent predictor for mental health outcomes: A cross-sectional study among Thai high-school students
Source: PLoS One. 2025 Jun 23;20(6):e0326432. doi: 10.1371/journal.pone.0326432 (PMC12184913; doi:10.1371/journal.pone.0326432)
Supplement: Attachment — Submitted filename: BuddhaImage_Reviewer comments.docx [file pone.0326432.s010.docx]

Additional Editor Comments:

Dear Authors,
Thanks for the revision. However, it was realised that you did not respond to the comment of Reviewer 8. Therefore, I will entreat you to respond to it.

Thank you for bring this to our attention.

I do not know whether you may still see it therefore, I am stating it here: "The paper seems to be modified based the reviewers' comments.
I just have a one comments about mother's skills and their health literacy that can have a mediated role in promoting the mental health of the school child people. So I suggest to check the following paper for more conceptualization of the mentioned issue:
Enabling mothers through improving mental health literacy and parenting skills
Biosocial Health Journal. 2024;1(1): 26-32. doi: 10.34172/bshj.4"

Thank you for the recommendation.

Please ignore the comment of Reviewer 9 this time or any other comments from a reviewer. Only address the one above (repost from Reviewer 8).

Reviewers' comments:

Reviewer's Responses to Questions

**Comments to the Author**

1. If the authors have adequately addressed your comments raised in a previous round of review and you feel that this manuscript is now acceptable for publication, you may indicate that here to bypass the “Comments to the Author” section, enter your conflict of interest statement in the “Confidential to Editor” section, and submit your "Accept" recommendation.

Reviewer #7: (No Response)

Reviewer #9: All comments have been addressed

2. Is the manuscript technically sound, and do the data support the conclusions?

The manuscript must describe a technically sound piece of scientific research with data that supports the conclusions. Experiments must have been conducted rigorously, with appropriate controls, replication, and sample sizes. The conclusions must be drawn appropriately based on the data presented.

Reviewer #7: Yes

Reviewer #9: Yes

3. Has the statistical analysis been performed appropriately and rigorously?

Reviewer #7: Yes

Reviewer #9: Yes

4. Have the authors made all data underlying the findings in their manuscript fully available?

The [PLOS Data policy](http://track.editorialmanager.com/CL0/http:%2F%2Fwww.plosone.org%2Fstatic%2Fpolicies.action%23sharing/1/010f0196448503ed-505d2776-dcab-4b55-844a-cda8f75a2adb-000000/nbZBK0UhGv8H-xe0xtXvy6OAf1oQMPzkYdZ4qrlVzeA=207) requires authors to make all data underlying the findings described in their manuscript fully available without restriction, with rare exception (please refer to the Data Availability Statement in the manuscript PDF file). The data should be provided as part of the manuscript or its supporting information, or deposited to a public repository. For example, in addition to summary statistics, the data points behind means, medians and variance measures should be available. If there are restrictions on publicly sharing data—e.g. participant privacy or use of data from a third party—those must be specified.

Reviewer #7: No

Reviewer #9: Yes

5. Is the manuscript presented in an intelligible fashion and written in standard English?

PLOS ONE does not copyedit accepted manuscripts, so the language in submitted articles must be clear, correct, and unambiguous. Any typographical or grammatical errors should be corrected at revision, so please note any specific errors here.

Reviewer #7: Yes

Reviewer #9: Yes

6. Review Comments to the Author

Please use the space provided to explain your answers to the questions above. You may also include additional comments for the author, including concerns about dual publication, research ethics, or publication ethics. (Please upload your review as an attachment if it exceeds 20,000 characters)

Reviewer #7: Review comments
General comments
I commend the Authors for the good review of the manuscript. I am of the opinion that the manuscript should be accepted for publication.

Authors should effect these changes as presented below
1. The Title should include the country where the study was conducted

Thank you. The authors have made the change.

2. The mean age of the respondents should be better presented as 16.35±0.96 years

Indeed, thank you. Change made.

3. The Journal, PLOS ONE uses square brackets for reference numbers instead of circular brackets

Acknowledged. Can this setting be changed by the copyeditor? The author is unable to make this change within Endnote despite repeated attempts.

Reviewer #9: This is a highly relevant and valuable study that has undergone multiple revisions, benefiting from these refinements to improve its clarity and scientific rigor, particularly concerning methodological aspects. Naturally, as with any research, there is always room for further enhancement. One potential avenue for improvement could involve a deeper exploration of the psychological mechanisms underlying meditation, particularly from a neuroscientific perspective. Understanding the neural processes at play during meditation, as well as identifying the key variables that influence its effectiveness, could provide valuable insights into how meditation can be optimized for different individuals and contexts.

Moreover, investigating the factors that contribute to a more or less beneficial meditation experience—both from the perspective of the practitioner and the technique itself—could offer a more comprehensive understanding of meditation's impact on cognitive and emotional well-being. Variables such as individual differences, meditation experience, environmental factors, and specific techniques employed could be examined in greater detail to determine their role in enhancing or diminishing the benefits of meditation. Additionally, exploring the interplay between meditation and neuroplasticity, stress regulation, and emotional resilience could further illuminate the long-term effects of meditation practices.

In this regard, the study could be further strengthened by making the introduction more explicit in its presentation of established scientific findings, rather than relying primarily on descriptive elements. A more thorough review of existing literature, incorporating recent advancements in neuroscience and psychology, could provide a stronger foundation for the study's hypotheses and methodologies. Moreover, an interdisciplinary approach, integrating insights from cognitive science, clinical psychology, and contemplative studies, could enrich the discussion and broaden the study’s implications. Additionally, a clearer articulation of how this study contributes to the broader scientific discourse on meditation would further enhance its significance and impact.

By integrating these refinements, the study would not only become more rigorous but also more insightful, paving the way for future research on the cognitive and neural mechanisms of meditation and its practical applications in promoting mental well-being. Expanding the scope to consider cultural and contextual differences in meditation practices could also provide valuable perspectives on its effectiveness across diverse populations.

Thank you for these many thoughtful recommendations. Regarding the biological and neural correlates of meditation, information was added in the early part of the introduction that reads as follows: “Neurobiological research has found that meditation may influence mental health through changes in the brain’s structure and its functioning. Mindfulness meditation is associated with increased activation in the prefrontal cortex and decreased activity in the amygdala (16, 17). These changes may support cognitive control and emotional reactivity, which may help with emotional regulation, resilience, and positive mental health outcomes.”
The authors have more clearly articulated how this research contributes to the conversation surrounding meditation. This addition can be found in the paragraph before “Materials and Methods,” it reads, “Mindfulness meditation has received considerable research attention, with much less research investigating multiple meditation styles within the same population. Thai people practice a wide array of meditation types embedded in cultural contexts that influence their meditation practice. This study expands the conversation about meditation by accounting for mindfulness meditation as well as lesser-known styles such as Buddha Image and Manomayiddhi. Adolescent mental health in Thailand can then be assessed in consideration of the many meditation types they may practice, as well as by accounting for their frequency of practice and sociodemographic influences.”

7. PLOS authors have the option to publish the peer review history of their article ([what does this mean?](https://track.editorialmanager.com/CL0/https:%2F%2Fjournals.plos.org%2Fplosone%2Fs%2Feditorial-and-peer-review-process%23loc-peer-review-history/1/010f0196448503ed-505d2776-dcab-4b55-844a-cda8f75a2adb-000000/zriXLOTiICnbKpqWBVBTXK870YU3Vrh8byalxGtGqIs=207)). If published, this will include your full peer review and any attached files.


If you choose “no”, your identity will remain anonymous but your review may still be made public.


**Do you want your identity to be public for this peer review?** For information about this choice, including consent withdrawal, please see our [Privacy Policy](https://track.editorialmanager.com/CL0/https:%2F%2Fwww.plos.org%2Fprivacy-policy/1/010f0196448503ed-505d2776-dcab-4b55-844a-cda8f75a2adb-000000/6qPYVF_t_-69jNeAP2IzfCfamNcNwC8yIkMDBayoJAU=207).

Reviewer #7: **Yes:** EDMUND NDUDI OSSAI

Reviewer #9: No
